# Gastropericardial fistula: getting to the heart of the matter

**DOI:** 10.1186/s12876-016-0510-8

**Published:** 2016-08-19

**Authors:** Vian Azzu

**Affiliations:** 1 Hinchingbrooke Hospital, Hinchingbrooke Park, Huntingdon, PE29 6NT UK; 2 Department of Medicine, University of Cambridge, Hills Road, Cambridge, CB2 0QQ UK

**Keywords:** Gastropericardial fistula, Pneumopericardium, Pericarditis, Gastric adenocarcinoma

## Abstract

**Background:**

Gastropericardial fistula is a rare life-threatening condition, being reported only 65 times in modern literature.

**Case presentation:**

A 67 year-old man who presented with weight loss, chest pain and epigastric pain was found to have pericardial effusion and pneumopericardium on computed imaging. Endoscopy and histology confirmed a gastric adenocarcinoma within a hiatus hernia, which had fistulated to the pericardium. His condition was complicated by pulmonary emboli and lobar infarction, all contributing to rapid deterioration and death.

**Conclusion:**

Review of all previously published cases reveals that factors which predict poorer prognosis are older age, cancer etiology and conservative management. Conversely, protective factors include younger age at presentation, previous gastroesophageal surgery or ulcers as an etiology, and aggressive procedural and surgical management. Although the diagnosis is viewed as largely fatal by many clinicians, operative management has contributed to a statistically significant reduction in mortality from 69 % in the pre-2000 era to 11 % in the post-2000 era. This study summarizes diagnostic methods and treatment interventions and prognostication in this rare condition.

## Background

Gastropericardial fistula is a life-threatening abnormal communication between the stomach and the pericardial sac. This condition is rare and its etiologies include previous gastric or esophageal surgery, ulcer perforation or cancer perforation. It usually occurs within a hiatus hernia and rarely occurs transdiaphragmatically. A review of all published cases reveals that aggressive procedural and surgical management has reduced mortality from 69 to 11 % in the last 15 years. This study details diagnostic methods and treatment interventions and prognostication in this rare condition.

## Case presentation

A 67 year-old male presented to his local hospital with six weeks of extreme lethargy. He complained of non-radiating chest and epigastric pain with associated breathlessness and anorexia. On further questioning he admitted to 25 kg weight loss over the previous six months. He reported a past history of empyema occurring decades previously.

Examination revealed normal heart sounds, an irregular tachycardia with a pulse of 100 beats/min, raised jugular venous pulse, widespread peripheral edema, vesicular air entry to lungs, no abdominal signs, and no lymphadenopathy.

Vital signs revealed a pyrexia of 39 °C, relative hypotension of 110/67 mmHg, pulse oximetry 95 % on air, a tachypnea of 26 breaths/min and normal urine output.

Laboratory investigations showed hemoglobin 57 g/L (125–160 g/L), MCV 71 fL (80–100 fL), white cell count 33.6 × 10^9^/L (4-11 × 10^9^/L), CRP 218 mg/L (<5 mg/L), sodium 127 mmol/L (135–145 mmol/L), potassium 5.9 mmol/L (3.5–5.5 mmol/L), creatinine 111 mmol/L (60–110 mmol/L), albumin 17 g/L (35–55 g/L), bilirubin 9 mg/L (0–17 mg/L), ALT 182 U/L (7–56 U/L), ALP 203 (44–107 U/L). Blood film demonstrated neutrophilia with left shift consistent with severe bacterial infection, and evidence of anemia including microcytosis, polychromasia, target cells and pencil red blood cells.

Initial treatment included transfusion of 4 units of packed red blood cells, treatment of heart failure with diuresis and of sepsis with intravenous broad-spectrum antibiotics (tazobactam/piperacillin).

**Fig. 1:**
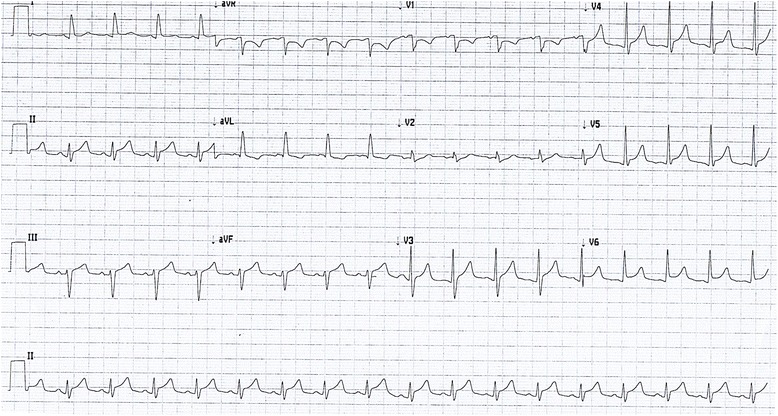
Electrocardiogram showing widespread ST elevation

**Fig. 2:**
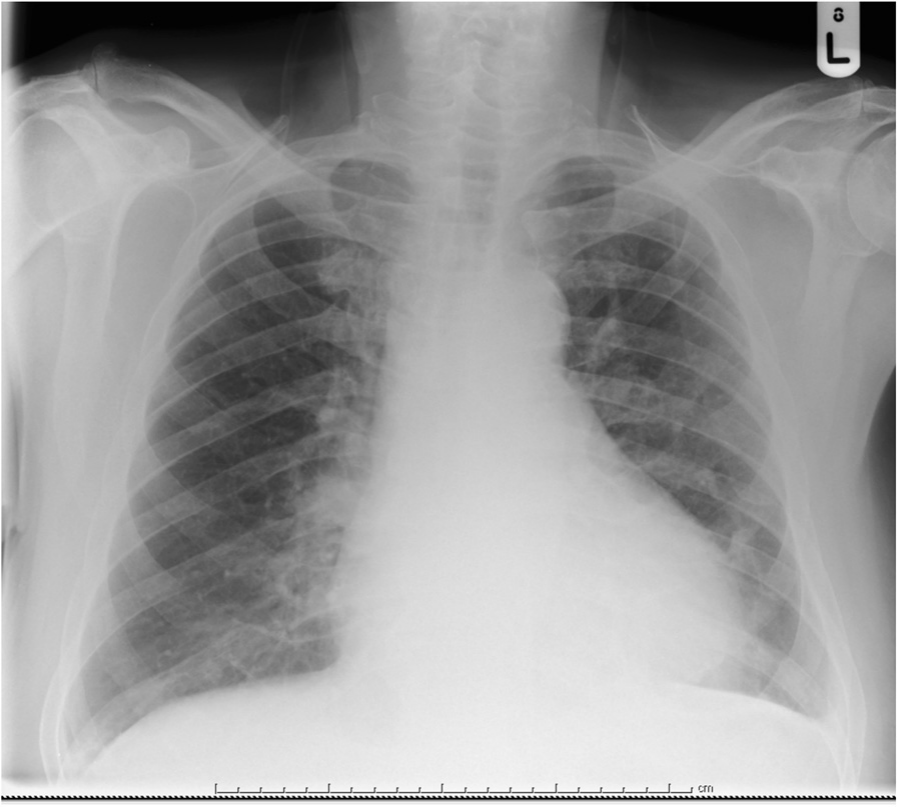
Chest radiogram showing small left-sided pleural effusion

Serial electrocardiograms showed sinus tachycardia with paroxysmal atrial fibrillation and widespread ST elevation of about 2 mm in leads II, III, aVF, V3-V6 (Fig. 1), which later normalized. Serial troponin I ultra over a 24 h period after presentation were 695, 538, 491 ng/L (<20 ng/L) respectively. It was thought this represented cardiac stress secondary to persistent tachycardia and profound anemia. Thoracic radiogram showed a small left-sided pleural effusion (Fig. 2).

Echocardiography was obtained and showed mild left ventricular dilatation with severe dysfunction and an ejection fraction of 25 %. There was also a 0.9 cm apical, 1.4 cm anterior and 1.3 cm posterior simple pericardial effusion without hemodynamic compromise or tamponade. For this reason, pericardiocentesis was not performed.

**Fig. 3:**
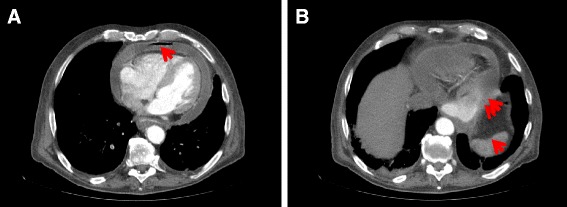
Computed tomogram showing **a** pneumopericardium (*arrow*) and pericardial effusion, and **b** mass within a hiatus hernia (*single arrow*) and likely area of fistulation (*double arrow*)

**Fig. 4:**
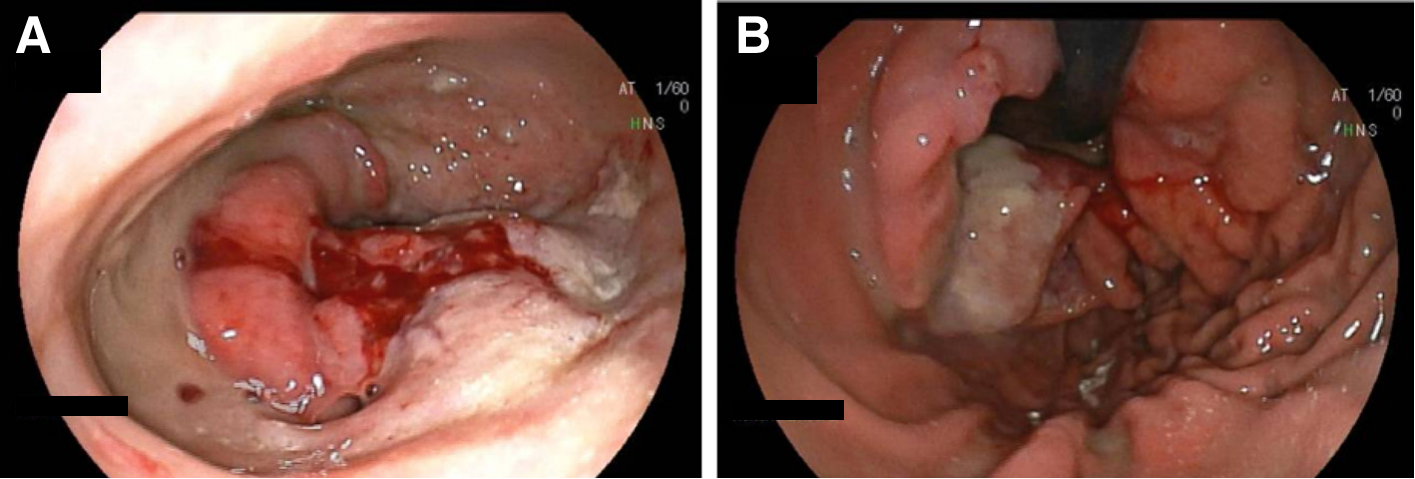
Gastroscopy showing **a** ulcerating mass within a hiatus hernia, and **b** view on endoscopic retroversion

Computed tomogram of chest abdomen and pelvis demonstrated right lower lobe pulmonary artery embolus with infarction of lung parenchyma, pneumopericardium and pericardial effusion (Fig. 3a), fixed large hiatus hernia with a mass (Fig. 3b), enlarged 18 mm celiac node and unremarkable appearances elsewhere. Subsequent gastroscopy confirmed a large hiatus hernia with a bleeding ulcerated gastroesophageal junction tumor (Figs. 4). Serosal breaching by this mass led to fistulation into the adjacent pericardium. Histological diagnosis was subsequently confirmed as poorly differentiated adenocarcinoma.

After an initial improvement with antibacterial therapy and hemodynamic stabilization, drainage of his contaminated pericardial cavity was considered, but the patient rapidly deteriorated with uncontrolled sepsis and multi-organ failure. Management of a complex case such as this requires multidisciplinary team discussion. It was felt that aggressive management of a cachectic man with a poor cancer prognosis and a multiple serious clinical conditions arising from this was not in his best interest as he was unlikely to survive intervention or surgery. These discussions included the patient and his family, and led to a palliative management approach.

## Literature search and discussion

The first case of pneumopyopericardium caused by subphrenic abscess due to gastric ulceration was described by Hallin in 1863 (cited by Pick) [1]. Nine similar cases were described subsequently [2]. The first description of a direct communication between the stomach and the pericardial sac was by Harp and colleagues in 1947 [3], in which they described the perforation of a gastric tumor into the pericardium. A comprehensive literature search identified a further 64 cases of gastropericardial fistula, of which one publication which was unobtainable [4].

Of the 65 cases including ours, 63 % of patients were men and 37 % were women giving a male:female ratio of 3:2. The mean age of presentation was 59 years (median 63 years). The modes of presentations included, in order of frequency, chest or left shoulder pain (66 % of cases), dyspnea (22 %), epigastric pain (20 %), fever (14 %) and dysphagia/vomiting/hematemesis/melena (12 %).

Interestingly, in some cases, patients had presented months or years earlier with these symptoms, but the diagnosis was not evident from baseline investigations [3, 5–8]. For patients ultimately diagnosed with gastropericardial fistula, typical investigational findings in the literature reflected our own case: widespread ST elevation consistent with pericarditis and pneumopericardium on plain or computed tomography. Radiographic investigations can be enhanced by the use of oral contrast medium, which reveals fistulous communication from the gastrointestinal tract into the pericardial space. The use of gastroscopy is considered controversial by many authors due to the theoretical risk of causing pneumopericardial tamponade, but no study to date has shown this to be of any clinical significance including our own.

The etiologies for gastropericardial fistula include previous gastroesophageal surgery, ulcer perforation, gastric cancer or a combination of the above. These etiologies and their frequencies are listed in Table 1. Prior operative risk factors for gastropericardial fistula were open or laparoscopic Nissen’s fundoplication, previous esophagectomy, hiatus hernia repair, bariatric surgery and other surgery as well as trauma. The mean time of presentation was 84 months after surgery (median 60 months).

**Table 1:** Gastropericardial fistula etiologies and their frequencies

Etiology of gastropericardial fistula	n	% of all etiologies
Gastroesophageal surgery and subsequent formation of an ulcer	20	31
Ulcer perforation	18	28
Previous surgery	18	28
- Open or laparoscopic Nissen’s fundoplication	- (6)	- (9)
- Bariatric surgery and other surgery	- (4)	- (6)
- Esophagectomy	- (4)	- (6)
- Hiatus hernia repair	- (2)	- (3)
- Trauma	- (2)	- (3)
Cancer perforation	6	9
Previous esophagogastrectomy for neoplasia with subsequent cancer recurrence	3	5

### Outcomes in gastropericardial fistula

Prior to the year 2000, average survival of those presenting with gastropericardial fistula was 31 %. The average age at presentation was 65 years and ulcer etiology was relatively common compared with subsequent years. All survivors had operative and interventional management including pericardiocentesis, pericardial/thoracic washout, pericardial window/pericardectomy, surgical fistula closure and ulcer repair or upper gastrointestinal tract repair. Of those who died, the majority were managed conservatively (64 %) and the remainder had an attempt at operative management (38 %). None of the patients who had cancer as an etiology survived.

Post-2000, average survival increased markedly to 89 %. The average age of presentation was lower at 54 years, and this may contribute to the lower mortality seen. Prior gastroesophageal surgery was more common as an etiology than in previous years. Only 6 % of survivors lived with only conservative management such as antibiotics and total parenteral nutrition; the remaining all had surgery to correct the gastropericardial fistula or procedural intervention such as pericardial drain. Of those who died, half were managed conservatively [9], half with pericardiocentesis [10, 11] and none with surgery. Of patients who had cancer as an etiology, 40 % survived and 60 % died. In the cancer patients who lived, intervention included total parenternal nutrition thus allowing for ulcer healing [12] and surgery with Ivor Lewis resection, lymphadenectomy, and pericardostomy [13]. These outcomes are summarised in Table 2.

**Table 2:** Outcomes in patients with gastropericardial fistula

	Pre-2000	Post-2000
Mean age at presentation	65 years	54 years
Predominant etiology	Ulcer (75 %)	Previous upper GI surgery (65 %)
Survival	31 %	89 %
Survivors/with operative intervention	100 %	94 %
Survivors/no operative intervention	0 %	6 %

Taking all 65 cases into account, 89 % of survivors had surgery whereas only 20 % of non-survivors did. In the group with perforating cancer, all patients who survived had surgery [13] or pericardiocentesis [12], whereas only half of those who died had surgical or procedural intervention [7, 10, 11]. Interestingly, those who survived were, on average, 13 years younger than those who did not (mean age of survivors 54 years, mean age of non-survivors 67 years) and this was mirrored in those with a cancer etiology (mean age of survivors 54 years, mean age of non-survivors 62 years). This analysis suggests that survival not only depends on younger age, but also on intervention/surgery, although the obvious confounder is that extremely unwell patients who are unlikely to survive surgery are not taken to theatre and have poorer outcomes, as was the case in our patient.

However, interpretation of the above data is complex and amounts to Level C evidence (expert opinion, series of case studies). Without exception, all cases recommend antibacterial therapy, optimization of fluid status and diversion of GI content away from the fistula plus nutritional support (Grade 1C evidence). The treatment effect of interventional procedures or surgery is less clear. It would be our conclusion that intervention (eg. pericardial drainage) should be considered in most patients order to temporize sepsis (Grade 2C), whilst more definitive management may be planned for a later time. Since patients fare better with surgery than without (Table 2), this approach should be strongly considered even in multi-morbid patients since this may be their only chance of survival (Grade 3C).

## Conclusions

Gastropericardial fistula is a rare diagnosis. Its early diagnosis is often obscured by its own rarity and the non-specific signs and symptoms associated with it. Its etiologies include previous upper GI surgery, perforating gastric ulcers and perforating cancers. The latter two occur more frequently in a heterotopic stomach such as in hiatus hernia. Patients may present with this diagnosis months before the ultimate presentation, and this diagnosis should be strongly suspected in patients with the above risk factors who present with chest/shoulder pain, dyspnea, pyrexia or upper GI symptoms. The most sensitive investigations are likely to be CT with oral contrast which may reveal a gastropericardial fistula and pneumopericardium, as well as echocardiogram which may reveal pericardial effusion. All patients should receive antibacterial/antifungal therapy, hemodynamic stabilization, diversion of GI contents away from the fistula as well as nutritional support. Patients should be considered for interventional procedures such as pericardial drainage. In patients not improving with the above approach, appropriate early surgical intervention is key to survival and should not be delayed. Clinicians should consider that a seemingly poor surgical candidate’s only chance of survival is operative management. Multidisciplinary team involvement is recommended for improved patient care in this complex and rare condition.
